# Comparison between three types of stented pericardial aortic valves (Trivalve trial): study protocol for a randomized controlled trial

**DOI:** 10.1186/1745-6215-14-413

**Published:** 2013-12-03

**Authors:** Kasra Azarnoush, Bruno Pereira, Christian Dualé, Enrica Dorigo, Mehdi Farhat, Andrea Innorta, Nicolas Dauphin, Etienne Geoffroy, Pascal Chabrot, Lionel Camilleri

**Affiliations:** 1 Heart Surgery Department, Clermont-Ferrand University Hospital, Clermont-Ferrand, France; 2 INRA, UMR 1019 Nutrition Humaine, F-63122 Saint Genès Champanelle, France; 3 Biostatistics Unit, Délégation Recherche Clinique & Innovation, Clermont-Ferrand University Hospital, Clermont-Ferrand, France; 4 Centre de Pharmacologie Clinique (Inserm CIC 501), Clermont-Ferrand University Hospital, Clermont-Ferrand, France; 5 Radiology Department, Clermont-Ferrand University Hospital, Clermont-Ferrand, France

**Keywords:** Stented pericardial aortic valves, Pericardial aortic valves, Hemodynamic performance

## Abstract

**Background:**

Aortic valve stenosis is one of the most common heart diseases in older patients. Nowadays, surgical aortic valve replacement is the ‘gold standard’ treatment for this pathology and the most implanted prostheses are biological ones. The three most implanted bovine bioprostheses are the Trifecta valve (St. Jude Medical, Minneapolis, MN, USA), the Mitroflow valve (Sorin Group, Saluggia, Italy), and the Carpentier-Edwards Magna Ease valve (Edwards Lifesciences, Irvine, CA, USA). We propose a randomized trial to objectively assess the hemodynamic performances of these bioprostheses.

**Methods and design:**

First, we will measure the aortic annulus diameter using CT-scan, echocardiography and by direct sizing in the operating room after native aortic valve resection. The accuracy of information, in terms of size and spatial dimensions of each bioprosthesis provided by manufacturers, will be checked. Their hemodynamic performances will be assessed postoperatively at the seventh day and the sixth month after surgery.

**Discussion:**

This prospective controlled randomized trial aims to verify and compare the hemodynamic performances and the sizing of these three bioprostheses. The data obtained may help surgeons to choose the best suitable bioprosthesis according to each patient’s morphological characteristics.

**Trial registration:**

ClinicalTrials.gov Identifier: NCT01522352

## Background

A critical aspect of aortic valve replacement is to achieve an optimal matching between the patient’s morphology and the implanted valve prosthesis. Specifically, the implanted prosthesis should not impair left ventricle ejection and this is even more crucial in cases where there is a small aortic annulus.

Pericardial bioprostheses have good hemodynamic performance because of their central opening and the flexibility of their leaflets. We already know that the durability of these pericardial bioprostheses is about 10 to 15 years [1,2].

At present, porcine bioprostheses are less well-performing than bovine pericardial ones [3] and among these, we focused on three bioprostheses offering high hemodynamic performance, especially for small aortic valves.

Since March 2010, a new pericardial aortic valve bioprosthesis (Trifecta valve, St. Jude Medical, Minneapolis, MN, USA) has been approved by the Food and Drugs Administration (FDA approval: St Jude Medical Trifecta Valve - P100029) and has recently received the CE mark. However, its hemodynamic characteristics still need to be compared with other bioprostheses already available on the market. Another bioprosthesis chosen for this trial is the Mitroflow valve (Sorin Group, Saluggia, Italy) [4]: it received the CE mark in July 2011 and it is characterized by an innovative phospholipid reduction treatment (PRT) expressively conceived to reduce the calcification process and, as a consequence, to improve its durability.

The third bioprosthesis is the Carpentier-Edwards Magna Ease valve (Edwards Lifesciences, Irvine, CA, USA) which has been designed by developing the renowned and highly performing Carpentier-Edwards PERIMOUNT valves. It allows easier implantation and its pericardial tissue is additionally treated to prevent calcification. This bioprosthesis received the CE mark in 2007 and FDA approval in 2009.

### Objectives of the Trivalve study

Each aortic valve has its own hemodynamic characteristics related to its geometry and each patient has their own morphology (weight, size, anatomy of aortic valve), as well as different physiological and pathophysiological conditions (ejection fraction, size and degree of calcification of the aortic annulus, degree of left ventricular hypertrophy and so on). Consequently, the choice of valve prosthesis and the surgical implantation technique are the only two directly adjustable variables; nonetheless, current literature does not provide clear differences among available bioprostheses.

The main objective of this study is to measure the hemodynamic performance of the three aortic bioprostheses: the Trifecta valve (St. Jude Medical, Minneapolis, MN, USA), the Mitroflow valve (Sorin Group, Saluggia, Italy), and the Magna Ease valve (Edwards Lifesciences, Irvine, CA, USA) Table 1.

**Table 1:** Bioprosthesis characteristics

**Bioprosthesis type**	**Manufacturer**	**Valve diameters (mm)**
Trifecta	St. Jude Medical, Minneapolis, USA	19 to 29
Mitroflow	Sorin Group, Saluggia, Italy	19 to 29
Carpentier-Edwards Magna Ease	Edwards Lifesciences, Irvine, USA	19 to 29

Secondary end points will focus on 1) comparison between the effective aortic orifice area measured by computerized tomography (CT)-scan and echocardiography and the intraoperative measurement performed by a flat-head candle; 2) comparison between the diameter of the aortic orifice measured by a flat-head candle and the size of the implanted bioprosthesis provided by the manufacturer; 3) testing the accuracy of information provided by the manufacturers about bioprosthesis diameters.

## Methods and design

**Figure 1:**
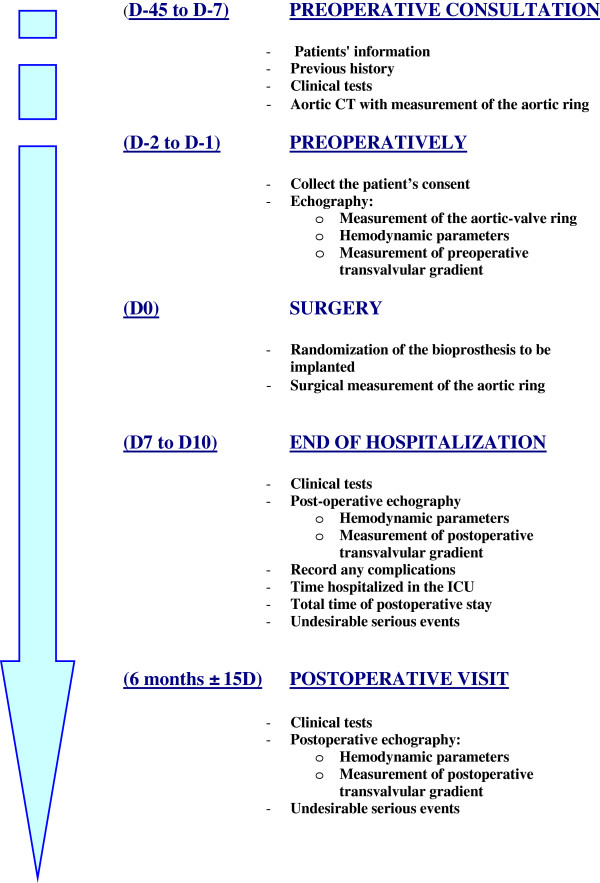
Study’s flowchart.

The Trivalve trial is a single-center, prospective, randomized trial. It will evaluate the short-term (six month) hemodynamic performance of three pericardial bioprostheses: the Trifecta valve (St. Jude Medical, Minneapolis, MN, USA), the Mitroflow valve (Sorin Group, Saluggia, Italy), and the Carpentier-Edwards Magna Ease valve (Edwards Lifesciences, Irvine, CA, USA), Figure 1.

ClinicalTrials.gov Identifier: NCT01522352.

### Patient’s enrollment and randomization

All patients scheduled for surgical aortic valve replacement by bioprosthesis will be screened according to inclusion and exclusion criteria (Table 2).

**Table 2:** Inclusion and exclusion criteria

	
**Inclusion criteria**	Isolated aortic valve replacement or associated with myocardial revascularization and/or tricuspid valve repair
	Age (> 18 years and < 85 years)
**Exclusion criteria**	Emergency surgery
	Surgery other than full sternotomy
	Heart transplantation
	Any procedure involving the aorta (such as Bentall procedure, surgery for dissection, and so on)
	Redo surgery
	Active infective endocarditis
	Associated mitral valve surgery
	Heart failure (ejection fraction < 40%) or preoperative cardiogenic shock
	Systolic pulmonary arterial pressure > 60 mmHg
	Patient’s protocol refusal
	Pregnancy
	Mentally handicapped patients, pre-existing psychiatric disease or addiction
	Advanced respiratory failure (forced expiratory volume in 1 second or vital capacity below 50% of the predicted)
	Severe renal failure
	History of allergy or intolerance to iodinated contrast infusion
	Patients living more than 100 km away from the investigation center

Patients who have given their signed informed consent to participate in this clinical trial will undergo, preoperatively, a CT-scan and a transthoracic echocardiogram to measure the aortic annulus. Included patients will be randomly allocated to receive one of the three bioprostheses, in a 1:1:1 ratio. When a patient is considered eligible and informed consent has been obtained, randomization will be performed automatically (using STATA software (StataCorp, College Station, TX, USA) before surgery by an independent biostatistician. No stratification will be done. The selected bioprosthesis will be implanted.

### Preoperative measurements

Preoperative CT-scan measured data, echocardiography and surgical measurements are shown in Table 3.

**Table 3:** CT-scan, echocardiographic and surgical measurements

	**Preoperative data**	**Operative data**	**Day 7 data**	**Month 6 data**
CT-scan				
Native aortic annulus (mm)	X			
Ascending aorta diameter (mm)	X			
Echocardiography				
LVTS (mm)	X		X	X
LVTD (mm)	X		X	X
LVPWT (mm)	X		X	X
IVST (mm)	X		X	X
LVSF (%)	X		X	X
LVEF (%)	X		X	X
Pulmonary arterial pressure	X		X	X
Cardiac output (L/min^-1^)	X		X	X
Cardiac index (L/min^-1^/m^-2^)	X		X	X
Mean transvalvular gradient (mmHg)	X		X	X
Maximal transvalvular gradient (mmHg)	X		X	X
Aortic orifice area (m^2^)	X		X	X
Aortic regurgitation degree (0–4)	X		X	X
Paravalvular leak			X	X
Surgery				
Internal aortic annulus diameter (mm)		X		
Estimated valve diameter (mm)		X		
Implanted valve diameter (mm)		X		

IVST: inter ventricular septum thickness, LVEF: left ventricular ejection fraction, LVPWT: left ventricle posterior wall thickness, LVSF: left ventricular shortening fraction, LVTD: left ventricle tele diastolic diameter, LVTS: left ventricle tele systolic diameter.

### Surgery

**Figure 2:**
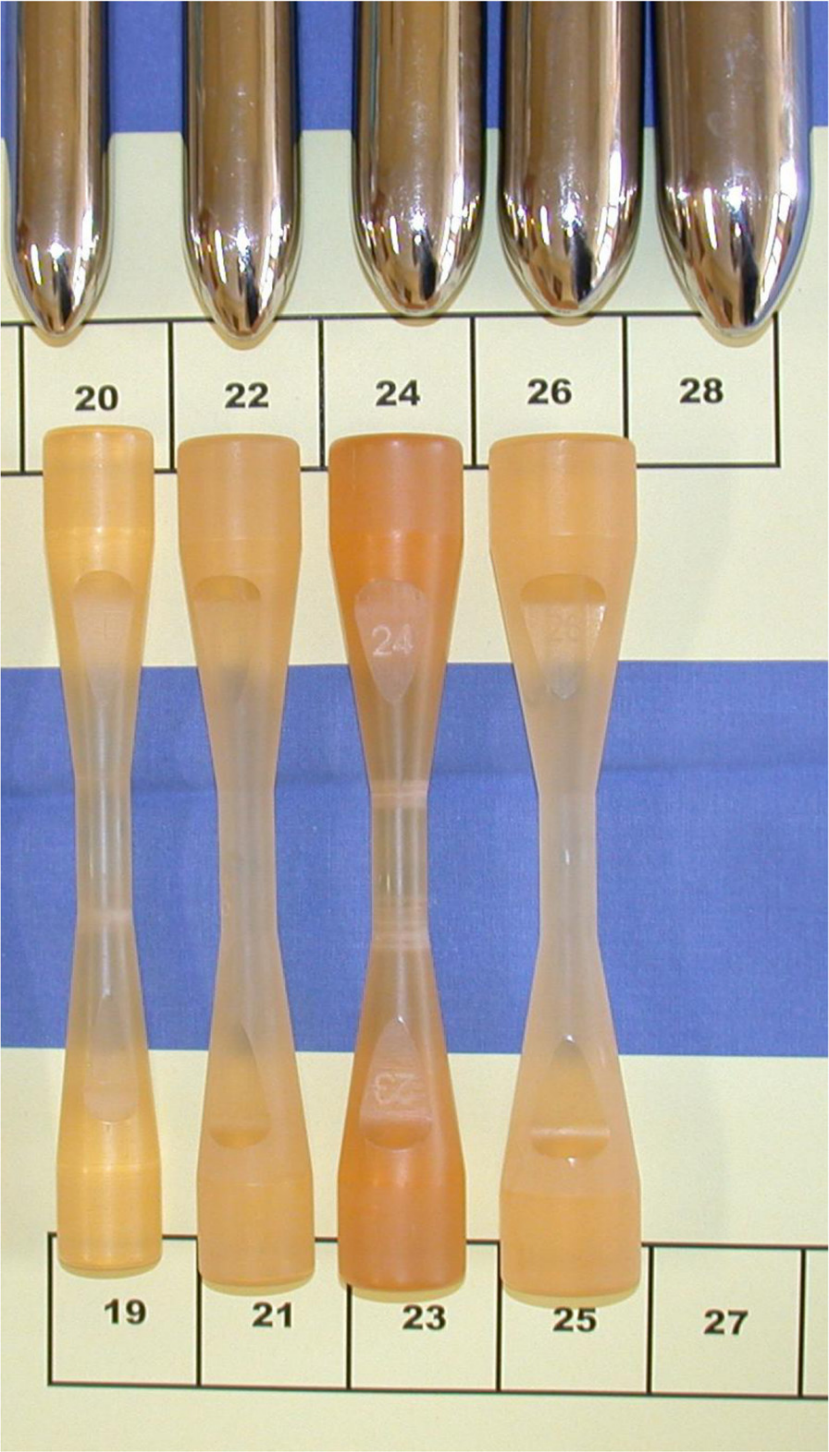
Importance of the use of flat-head candles to measure the aortic ring (bottom of the picture) compared to Hegar dilators.

During surgery, the aortic valve of the patient will be completely removed and the aortic annulus measured using a flat-head candle. This universal candle has been specifically designed to give a single objective value of the internal diameter of the aortic annulus. The Hegar dilators will not be used as its round shape and arched aerodynamics overestimate the size of the annulus by applying opening force to its passage (Figure 2). All measurements, early postoperative complications and reoperations for bleeding will be recorded in the operative report and in the case-report form.

### Postoperative endpoints

All preoperative echocardiography collected measures will be reassessed at day seven and month six after surgery in addition to maximal and mean transvalvular gradients (mmHg). ICU, total hospital stay and any other postoperative complications will be recorded in the postoperative report and in the case-report form.

The primary endpoint is the mean transvalvular gradient (mmHg) six months after surgery. All secondary endpoints are indicated in Table 4.

**Table 4:** Primary and secondary endpoints

	
**Primary endpoint**	Mean transvalvular gradient (mmHg), six months after surgery
**Secondary endpoints**	Effective aortic orifice diameter measured by CT-scan and echocardiography compared with the surgical data (mm)
	Mean transvalvular gradient (mmHg), at day seven after surgery
	Aortic bioprosthesis orifice area (m^2^), six months after surgery
	Diameter of the aortic orifice measured by a flat-head candle compared with the size of the implanted bioprosthesis (mm)

### Statistical considerations

#### Sample size estimation

The estimation of the number of patients required was considered by using previous data provided by the manufacturers on patients who had cardiovascular surgery which showed the mean postoperative gradients (mmHg) for the three studied types of valve (data not published). A minimum difference (δ) of 4 mmHg could be expected between the three types of valve for the most relevant diameters (21 and 23 mm). As the information concerning statistical variability was not provided in this document, the standard deviation (σ) was estimated on the basis of data from 103 patients observed in our center (84 with the Edwards Ease prosthesis, 19 with a Mitroflow diameter of 21 or 23 mm): σ = 5.8. Thus, for a type 1 error α = 0.05 (two-sided), a 90 %-power, δ = 4 and σ = 5.8, 44 subjects per group are needed. Taking into account multiple comparisons between the three randomized groups, 55 patients per group will be included (165 patients in total).

### Statistical analyses

All analyses will be performed using STATA v11 (StataCorp, College Station, TX, USA). A two-tailed *P*-value of 0.05 will be considered statistically significant. All analyses will be performed on an intention-to-treat (ITT) basis. The number of included patients and the rate of inclusions will be presented over time for each group. The patients will be described and compared between groups at baseline according to the following variables: compliance with eligibility criteria, epidemiological features, clinical features (including echocardiographic) and biological characteristics. The comparison concerning the postoperative means of the transvalvular gradients (measured by echocardiography at six months post surgery) between the three groups will be evaluated using ANOVA followed by the Tukey-Kramer *post hoc* test, or the Kruskal-Wallis nonparametric test if conditions of ANOVA are not met (homoscedasticity studied by Bartlett’s test and normality verify by Shapiro-Wilk) followed by Dunn’s test as appropriate. Comparisons between the groups will be realized systematically 1) without adjustment and 2) when appropriate, after adjustment (by multivariate linear regression model) on factors whose distribution could be unbalanced between the arms despite randomization. Quantitative secondary endpoints (for example hemodynamic data, CT-scan, in-hospital stay) will be analyzed as described above. Categorical parameters (that is, proportion of reoperations) will be compared between the groups using the chi-squared test or Fisher’s exact test, when necessary. To assess the relationships between the quantitative parameters (comparison between aortic orifice measurements by echocardiography, CT-scans, intraoperative measurement using the flat- candle versus the size of implanted valve prosthesis given by the manufacturer), the correlation coefficients (Pearson or Spearman), the Lin concordance coefficient and the intra-class coefficient (ICC) will be calculated. Later on, an ANCOVA could be proposed to consider group effect. The intra-group comparisons related to the quantitative criteria (hemodynamic data by echocardiography on preoperative period and at six months) will be made using paired the ANOVA or Wilcoxon test. Finally, to avoid bias induced by the presence of missing data, particularly with regards to the mean postoperative transvalvular gradient at six months (lost at follow-up or deaths), the primary analysis (ITT with imputation data determined according to quantity and type of missing data) will be completed on a second time by a per-protocol analysis.

### Expected adverse events

These three prosthetic valves are made of three layers of fixed bovine pericardium assembled on a support (stent). They are then fixed in a glutaraldehyde solution and conditioned in a sterile manner. A correctly sized and implanted valve leads to very few complications. They have an average lifespan of > 10 years when implanted in patients aged > 65 years [1,2]. The expected adverse events of these bioprostheses are those of usual heart valve replacement surgery on cardiopulmonary bypass and mortality can be predicted by Euroscore 2 [5], which is systematically calculated for all our patients. Postoperative adverse events will be evaluated according to the Clavien-Dindo classification for surgical complications [6].

### Funding

Edwards Lifesciences (Irvine, CA, USA), St. Jude Medical (Minneapolis, MN, USA), and Sorin Group (Saluggia, Italy) gave a contribution of €5,000 each and the Hospital Clinical Research Program (PHRC) of the French Ministry of Health contributed an amount of €15,000 for the realization of this study.

## The status of this trial

This trial has been actively recruiting patients since March 2012. The French Committee on Human Research (CPP Sud-Est VI) consented to this trial on 17 January 2012. Patients give their informed consent before being enrolled in this study. Agreement from the French Competent Authority (ANSM) was obtained on 23 June 23 2011. The completion date for this study is estimated as December 2014. The ClinicalTrials.gov identifier is NCT01522352.

## Discussion

Several studies have emphasized the importance of valve prosthesis hemodynamic performance [7,8], but none has taken into account more than one or two bioprostheses at a time [9,10]. Thus, we strongly believe that a randomized trial, with no direct conflicts of interest with industry, is mandatory to compare the three bioprostheses most commonly implanted in France and all around the world.

Our secondary objective is to compare the reliability of preoperative CT-scan and echocardiography used to assess the size of the aortic annulus in comparison to the surgical measurement. Both techniques (CT-scans and echocardiography) are already successfully used to predict the correct size of the aortic annulus before trans-catheter valve implantation procedures [11]. Another secondary objective, once we have assessed the surgical diameter of the aortic annulus intraoperatively, is to verify the reliability of the prosthesis size provided by the manufacturer. This last point is interesting as it aims to clarify an issue often debated by surgeons who complain to manufacturers that they over- or underestimate valve sizes. Precise information on the size of the implanted bioprosthesis compared to the real dimensions of the aortic annulus will guide cardiac surgeons to choose between these three bioprostheses according to the patient’s morphological characteristics.

## Abbreviations

ANSM: Agence nationale de sécurité du médicament; CE: Conformité Européenne; CPP: Comité de protection des personnes; CT: Computerized Tomography; FDA: Food and drugs administration; ICC: Intra-class coefficient; ITT: Intention-to-treat; mmHg: Millimeter of mercury; PRT: Phospholipid reduction treatment.

## Competing interests

The authors declare that they have no competing interests other than mentioned in this manuscript. Edwards Lifesciences (Irvine, CA, USA), St. Jude Medical (Minneapolis, MN, USA), and Sorin Group (Saluggia, Italy) gave a contribution of €5000 each and the Hospital Clinical Research Program (PHRC) of the French Ministry of Health contributed an amount of €15,000 for the realization of this study.

## Authors’ contributions

KA: conception, design, surgical management and writing of the manuscript. BP: conception, design and statistical management. CD: conception, design and statistical management. ED: critical revisions of the manuscript. MF: conception, design and surgical management. AI: conception, design and surgical management. ND: conception, design and echocardiography. EG: conception, design and echocardiography. PC: conception, design and CT-scan. LC: conception, design, surgical management, critical revisions and final approval. All authors read and approved the final manuscript.
